# Optimization of *in vitro* culture conditions of common carp germ cells for purpose of surrogate production

**DOI:** 10.3389/fvets.2022.1036495

**Published:** 2022-10-14

**Authors:** Xuan Xie, Roman Franěk, Martin Pšenička, Fan Chen, Vojtech Kašpar

**Affiliations:** ^1^Department of Gynecology & Obstetrics, Xijing Hospital of Airforce Military Medical University, Xi'an, China; ^2^Research Institute of Fish Culture and Hydrobiology, South Bohemian Research Center of Aquaculture and Biodiversity of Hydrocenoses, Faculty of Fisheries and Protection of Waters, University of South Bohemia in Ceské Budějovice, Ceské Budějovice, Czechia; ^3^Department of Genetics, The Silberman Institute, The Hebrew University of Jerusalem, Jerusalem, Israel; ^4^Department of Neurosurgery, Tangdu Hospital of Fourth Military Medical University, Xi'an, China

**Keywords:** common carp, germ cell, germ cell culture, Sertoli cell, feeder cell

## Abstract

Common carp (*Cyprinus carpio*) is the fourth most-produced fish species in aquaculture and frequently used model species with significant effort invested in development of biotechnological applications. In present study, we attempted to establish an *in vitro* germ cell culture condition for short term cell culture, which could facilitate further applications such as surrogacy or gene manipulation. Basal media and different types of feeder cells were investigated to optimize carp germ cell culture condition to favor maintenance of mitotic proliferation. Results indicated that germ cells cultured with hESC media and RTG2 cell line as feeder possessed significantly higher proliferation and survival rate compared to that cultured with StemPro media and Sertoli cell line as feeder. In addition, we compared two dissection strategies to compare risk of cell culture contamination and body cavity was open from dorsal part or from ventral part. As a result, carp open from the dorsal side can minimize the risk of contamination. In summary, this is the first study to optimize the cultivation of germ cells in common carp. This opens up new opportunities for the application of specific techniques in the breeding of those species with high commercial value and frequent use as a model fish. Results obtained in this study are important for implementation of new strategies in common carp breeding, conservation of genetic resources, restoration of lines or development of clonal and isogenic carp lines.

## Introduction

Common carp (*Cyprinus carpio*) is the fourth most cultured fish species in aquaculture, with production reaching 4,411,900 metric tons in 2019 (FAO Fisheries Statistics). This species is reared both in Europe and Asia, with significant effort invested in establishing breeding programs (1–3) or programs for conservation of genetic resources (4, 5). Genetic resource preservation resulted in development of *in vitro* conservation strategies for sperm (6, 7), germ cells (8), and development of surrogate production technology (9). Common carp is not only a species of commercial interest, but popular model species as recognized by OECD guidelines. Although cryopreservation protocols have been established to provide ways for restoration of developed breeds, utilization of cryopreserved milt has not reached commercial levels for production and breeding programs. Unfortunately, ovarian follicles have been damaged after cryopreservation (10, 11). Thus, surrogate production based on transplantation of carp germ cells into the body cavity of goldfish (*Carassius auratus*) shows promising results, providing a possibility for conservation of the female lineage.

Fish germ cells have the unique ability to self-renew as well as to differentiate into other germ cell stages. Both type A spermatogonia and oogonia show high sexual plasticity even after maturation of the donor (12). This specific attribute allows them to be used for biotechnological applications, specific breeding strategies, or to support the conservation of genetic resources (13). Germ cells cultured *in vitro* can be transplanted when populations are declining, or when limited numbers of germ cells are obtained from suitable or available donors. As well, this technology is available when stem cell purification shows low efficacy, or when cells are damaged by enzymatic dissociation. In recent years, *in vitro* culture of germ cells was established for some model species, including zebrafish (*Danio rerio*) (14), medaka (*Oryzias latipes*) (15), rainbow trout (*Oncorhynchus mykiss*) (16), or sturgeon *spp*. (*Acipenser spp*.) (17). Therefore, in present study, we aimed to establish *in vitro* culture conditions of carp germ cells using different types of culture medium and to investigate whether feeder cells are essential for carp germ cell proliferation.

## Materials and methods

### Animal ethics statement

Experiments were conducted at the Genetic Fisheries Center, Faculty of Fisheries and Protection of Waters (FFPW) in Vodnany, Czech Republic. The facility is authorized to perform the described manipulations and to perform experiments on animals (Act no. 246/1992 Coll., ref. number 16OZ19179/2016–17214). The methodological protocol of the current study was approved by the expert committee of the Institutional Animal Care and Use Committee of the FFPW, according to the law on the protection of animals against cruelty (reference number: MSMT-6406/119/2). The study did not involve endangered or protected species. Authors of the study hold certificates of professional competence for designing experiments and experimental projects under Section 15d (3) of Act no. 246/1992 Coll. on the Protection of Animals against Cruelty.

### Fish disinfection and dissection

Common carp used for present study were cultivated at the Faculty of Fisheries and Protection of Waters, University of South Bohemia. Gonads were collected from 6 to 9 month old carp (length ranged from 15 to 20 cm and total weight ranged from 0.15 to 0.20 kg). Carp were anesthetized by 0.05% 3-aminobenzoic acid ethyl ester methanesulfonate-222 (MS-222, Sigma, USA) until no gill movements where observed and there was no response to a tail pinch. Fish were bleed by cutting the gills and then the whole body was disinfected with 70% ethanol for 1 min and briefly dried on a paper towel. Based on previous germ cell transplantation studies, and to avoid contamination, we opened the fishes body cavity from the dorsal and ventral side, respectively. In brief, carp were cut from the dorsal side with a longitudinal cut using sterile scalpels and forceps, and the body cavity was open from dorsal side. On the other hand, we cut fish along belly from ventral side, exposed abdominal cavity and remove gut. In total, 30 carp were dissected by each of these methods. Gonads were gently collected by forceps. Each gonad was washed twice with phosphate-buffered saline (PBS; Gibco) containing 50 μg/mL ampicillin, 200 U/mL penicillin, 20 μg/mL streptomycin (all from Sigma-Aldrich, pH 8.0) in petri dishes.

### Testicular cell preparation

Dissociation of carp testicular cells was performed according to Xie et al. (17). Gonads were washed in PBS and minced into ~1 mm^3^ aliquots. Next, pieces of tissue were dissociated by 0.25% trypsin (Gibco) with 0.05% fetal bovine serum (FBS) and 40 U/mL DNase I (Sigma-Aldrich) in PBS at 24 °C for 2 h. The digestion was stopped by L-15 medium supplemented with 20% (v/v) fetal bovine serum (FBS), filtered through a 40 μm pore-size nylon screen and centrifuged at 300 g. The cell pellet was resuspended in PBS.

### Germ cell enrichment and culture condition optimization

After digestion, spermatozoa were eliminated by Ficoll-Paque PLUS density gradient media (GE Healthcare). Gradient separation was performed by centrifugation at 500 g for 30 min at room temperature. Enriched cells were seeded in a 6-well plate at a concentration of 1,200 cells/mm^2^. Cells were cultured at 21°C without CO_2_. After 4–5 days of culture, germ cells were enriched by differential plating and re-seeded on feeder cells at a concentration of 800 cells/mm^2^ in a 48-well plate. Components of culture medium are showed in Table 1. To remove somatic cells and to enrich germ cells, differential plating was performed for every passage.

**Table 1:** Components of cell culture medium.

**Components**	**Medium**	
Basal media	hESC	Stempro-34
Fetal bovine serum (10%)	+	+
Bovine serum albumin (2.50%)	+	+
2-mercaptoethanol (55 μm)	+	+
Basic fibroblast growth factor (bFGF, 10 ng/mL)	+	+
Glial cell line-derived neurotrophic factor (GDNF, 25 ng/mL)	+	+
leukemia inhibitory factor (LIF, 25 ng/mL)	+	+
Ascorbic acid (50 μm)	+	+
Chemically defined lipid concentrate (0.10%)	+	+
hESC supplement (2%)	+	+
Carp serum (1%)	+	+
non-essential amino acids (NEAA, 1%)	+	+
B-27 (1x)	+	+
Penicillin (50 μg/mL)	+	+
Ampicillin (50 μg/mL)	+	+
Streptomycin (50 μg/mL)	+	+

### Feeder cell preparation

In present study, RTG-2 and sertoli cell line were utilized as feeder cells. RTG-2 was purchased from ATCC (Summit Pharmaceuticals International, Tokyo, Japan) and maintained according to the instructions, while the sertoli cell line was derived from rainbow trout and maintained >50 passages (18). To use these cell lines as feeder cells for germ cell culture, proliferation activity was inhibited by treatment with 10 μg/mL mitomycin C for 6 h and cells were seeded onto 0.1% gelatin-coated plates at a concentration of 1200 cells/mm^2^ in a 48-well plate.

### Germ cell proliferation assay

To detect germ cell proliferation, a EdU incorporation assay was performed by adding 10 μM EdU (Sigma-Aldrich, USA) to the culture medium during the final 24 h of culture. EdU staining and 4,6-diamidino-2- phenylindole (DAPI) staining were performed with EdU Cell Proliferation Kit for Imaging (BCK-EdU647, baseclick, Germany) according to kit instructions. Then the cells were incubated 1 h at room temperature with DDX4 (vasa) rabbit polyclonal antibody (dilution 1:300, final concentration 1.8 μg/mL, GTX116575, GeneTex) and exposed for 1 h with secondary antibody anti-rabbit IgG–fluorescein isothiocyanate (FITC; F0382, Sigma, dilution 1:50) followed by staining with DAPI (3 ng/mL). The ratios of vasa-EdU-positive cells/total cells were calculated. Cell proliferation assays with EdU were performed at 7, 14, 21, and 28 days of culture at 21°C.

### Histology

The maturational stage of each testis was determined using histological techniques. Testicular tissue from donor common carp was fixed overnight in Bouin's fixative and processed for paraffin sectioning and stained with Masson trichrome stain. Histological sections were photographed using a microscope with mounted camera (Olympus BX61).

### Statistical analyses

The results were expressed as a ratio against control as mean ± SEM. Significance was determined with two-way analysis of variance (ANOVA) followed by Tukey's tests. Alpha was set at 0.05.

## Results

### Dissection of donor carp specimens

**Figure 1:**
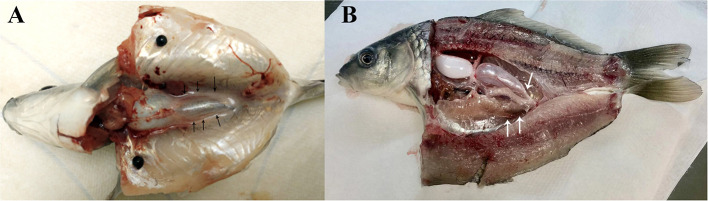
Common carp dissection illustration. **(A)** Carp dissected from ventral side. **(B)** Carp dissected from dorsal part. Gonads are indicated by arrowheads.

In present study, two dissection strategies were applied to avoid contamination of common carp gonads and 30 individuals were dissected in each group (Figure 1). Results showed that 24 out of 30 fish were contaminated when the body cavity was approached from ventral side. However, when the body cavity was open from the dorsal side, only 2 out of 30 fish showed signs of contamination.

### Optimalization of basal culture condition of carp germ cells

**Figure 2:**
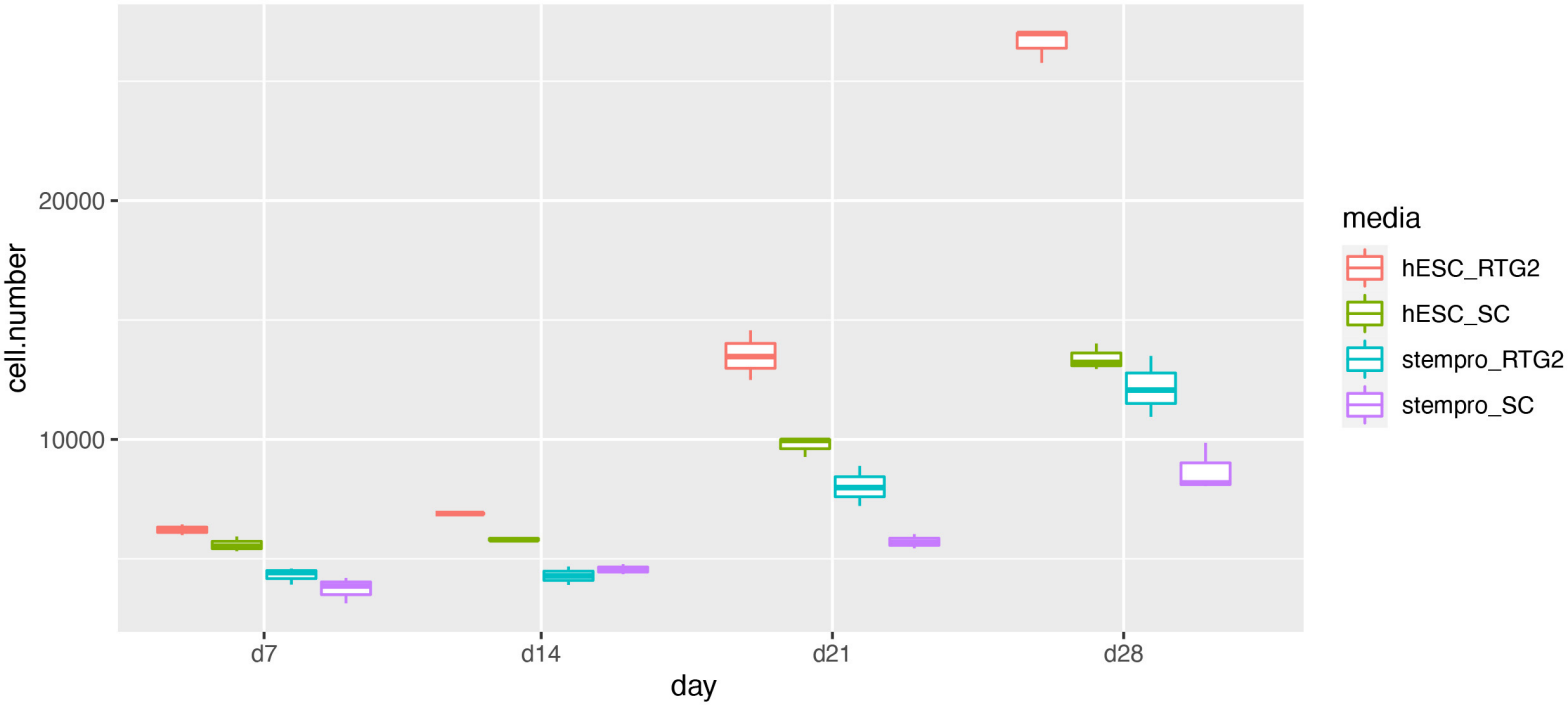
Effects of somatic cells on mitotic activity of common carp germ cells. The figure shows mean ± SEM (*n* = 3) of total germ cell number under hESC and stempro media with RTG2 and Sertoli cell line (SC). Cell number was count on days 7, 14, 21 and 28 of culture. Data are shown as the mean ± SEM (*n* = 3).

**Figure 3:**
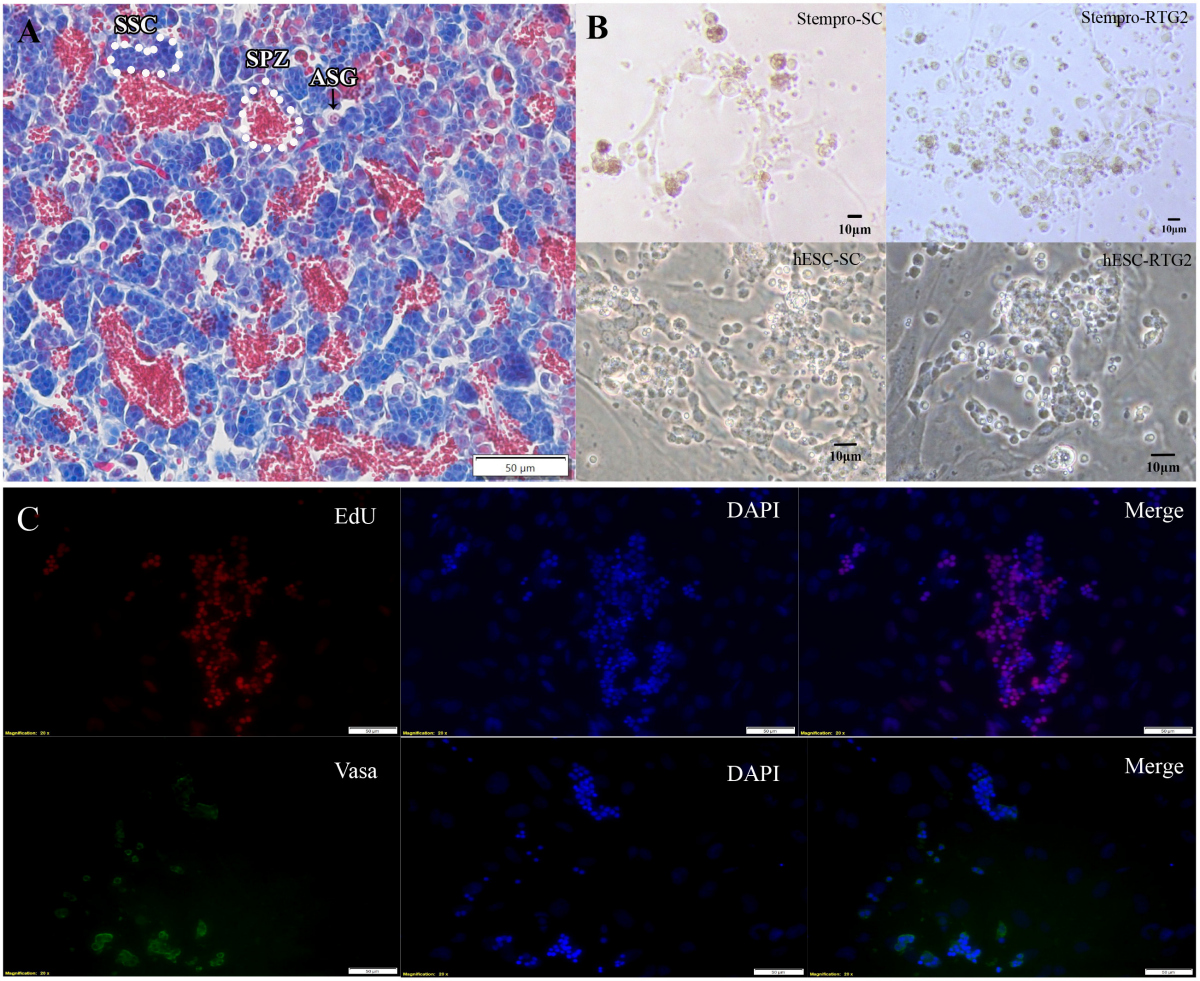
**(A)** Histological observation of common carp gonads. Various germ cell populations are indicated by arrowhead or white broken lines. **(B)** Morphology of cells cultured under different condition. **(C)** Immunocytochemistry of cultured cells at day 14. hESC-SC, hESC medium with Sertoli cells; hESC-RTG2, hESC medium with RTG2 cells; stempro-SC, stempro medium with Sertoli cells; stempro-RTG2, stempro medium with RTG2 cells. Less differentiated germ cells are blue, more differentiated are red, some spermatozoa are in the lumens already.

According to histological observation, testes contained type A spermatogonia, type B spermatogonia, spermatocytes, and few developing spermatids (Figure 1). We identified an optimal culture medium for carp germ cells by comparing two different basal culture media. Enriched carp germ cells were cultured in two different types of medium (Table 1) and the number of germ cells were monitored on after 7, 14, 21, and 28 days of incubation. Two-way ANOVA, using quantity of germ cells as the dependent variable, and media and feeder cells as main effects, revealed significant variation (*p* < 0.001) in the proliferation of germ cells for both factors (Figure 2). In addition, there was a significant interaction between media and feeder cell types (*P* = 0.049 < 0.05). For groups with hESC and StemPro media, significantly greater proliferation was shown in those cultured with hESC media. The most significant increase of germ cells was observed with hESC culture medium and RTG2 feeder. Germ cell propagation was observed throughout the 28 day test period. Cells cultured with RTG2 formed clumps and expanded, whereas cells lost their shape and turned apoptotic when cultured with Sertoli cells (Figure 3A).

In this study, we also assessed whether Sertoli and RTG2 cell lines could serve as feeder cells to improve germ cell proliferation. After 2 weeks, newborn cells cultured with the RTG2 cell line were detected through EdU assays, indicating that cells cultured with the RTG2 cell line had better proliferation activity (Figure 3B). After 2 months, we still could observe germ cell clumps according to vasa antibody immunocytochemistry (Figure 3C).

## Discussion

The gut of common carp carries symbiotic bacteria. Thus, removing gut contents during germ cell processing may release bacteria into the body cavity, which can contaminate germ cell cultures (19). To address this issue, we have compared two methods to dissect and open the body cavity of common carp. Our results revealed that dissection of fish from the dorsal part enabled us to avoid contamination.

A combination of internal and extrinsic mechanisms control fish germ stem cell survival, self-renewal, and differentiation (20–22). Somatic cells, particularly sertoli cells, assist in germ cell development and provide growth factors that positively affect germ cell proliferation and destiny. Germ cells are capable of autonomously controlling the differentiation pattern. For germ stem cell proliferation and differentiation, as well to start meiosis, gonadal somatic cells must produce growth factors (23, 24). Therefore, in present study, we investigated whether RTG2 and Sertoli cell lines can work as feeder cells to support germ cell proliferation. Our results indicated that in comparison with Sertoli cells, RTG2 could significantly improve carp germ cell growth. In addition, it turned out that germ cells performed better proliferation rate when it cultured with hESC media than stempro- 34 media. Applications of hESC were also reported in germ cell culture of human (25) and rainbow trout (18). It probably indicated that compared with stempro- 34 media, stempro hESC media are more capable of domestication of germ cells.

During fish spermatogenesis, germ cell survival and development depend on constant close contact with Sertoli cells (26). Sertoli cell are involved in the regulation of spermatogonial stem cell self-renewal and differentiation by secreting growth factors. In zebrafish, Igf3 is expressed in Sertoli cells and promotes differentiation and proliferation by activating β-catenin signaling in the germ cells. Amh, a member of the TGF-β (transforming growth factor-beta) superfamily produced by Sertoli cells, exerts an inhibitory role on spermatogonial self-renewal and germ cell differentiation in the zebrafish testes. In addition, GDNF has been proven in many fishes produced by Sertoli cells plays an important role in SSC self-renewal (27).

Interestingly, when RTG2 was used as feeder cells they could promote more germ cell propagation than Sertoli cells. Especially during the later stages of culture, the growth rate of germ cells decreased when cultured with Sertoli cells. This phenomenon was probably because the Sertoli cell line utilized in this study was derived from rainbow trout. Gdnf is not produced as an autocrine SSC niche factor in rainbow trout testes, unlike in mammals, as evidenced by the fact that it is expressed in germ cells from spermatogonia to spermatocytes but not in Sertoli cells (28). To overcome this issue, extra human recombined GDNF was supplemented in culture medium. However, germ cell proliferation rate did not remain as expected. It will be of interest decipher differences in growth factors secreted by RTG2 and Sertoli cell lines in the future. Moreover, germ cell transplantation is expected to be applied to investigate the germ cell stemness after prolonged culture.

In conclusion, a short-term carp germ cell *in vitro* culture condition has been established by optimizing the dissection method, basal culture mediums, and types of feeder cells. To date, cells could be maintained > 2 months, which is suitable for several research applications. This is the first report of successful *in vitro* germ cell culture in common carp, species important for aquaculture production as well as important model species in basic and applied science. Development of successful *in vitro* germ cell culture enables number of applications in surrogate production such as development and effective production of isogenic fish lines or production, restoration of lines or breeds from few available individuals or development of lines based on reported superior genotypes. In the future, it could be of interest to prolong carp germ cell proliferation and to investigate their cell capacity by transplantation.

## Data availability statement

The raw data supporting the conclusions of this article will be made available by the authors, without undue reservation.

## Ethics statement

The animal study was reviewed and approved by Institutional Animal Care and Use Committee of the FFPW.

## Author contributions

Conceptualization, supervision, and validation: XX and VK. Data curation and formal analysis: XX, VK, and RF. Funding acquisition and investigation: VK and MP. Methodology: XX, RF, MP, and VK. Project administration: VK. Resources: XX and RF. Visualization and writing—original draft: XX, VK, and FC. Writing—review and editing: all authors. All authors contributed to the article and approved the submitted version.

## Funding

This work was supported by National Agriculture Agency project number QK1910428, Czech Science Foundation project 22-01781O and 22-31141J, by the Ministry of Education, Youth and Sports of the Czech Republic and project Biodiversity (CZ.02.1.01/0.0/0.0/16_025/0007370). This project has received funding from the European Union's Horizon 2020 Research and Innovation Programme under grant agreement and No. 871108 (AQUAEXCEL3.0). This output reflects only the authors' view and the European Union cannot be held responsible for any use that may be made of the information contained therein.

## Conflict of interest

The authors declare that the research was conducted in the absence of any commercial or financial relationships that could be construed as a potential conflict of interest.

## Publisher's note

All claims expressed in this article are solely those of the authors and do not necessarily represent those of their affiliated organizations, or those of the publisher, the editors and the reviewers. Any product that may be evaluated in this article, or claim that may be made by its manufacturer, is not guaranteed or endorsed by the publisher.
